# Facial Emotion Recognition in Schizophrenia

**DOI:** 10.3389/fpsyt.2021.633717

**Published:** 2021-05-04

**Authors:** Zhiyun Gao, Wentao Zhao, Sha Liu, Zhifen Liu, Chengxiang Yang, Yong Xu

**Affiliations:** ^1^Department of Psychiatry, First Hospital/First Clinical Medical College of Shanxi Medical University, Taiyuan, China; ^2^Department of Humanities and Social Science, Shanxi Medical University, Taiyuan, China; ^3^Shanxi Key Laboratory of Artificial Intelligence Assisted Diagnosis and Treatment for Mental Disorder, First Hospital of Shanxi Medical University, Taiyuan, China

**Keywords:** schizophrenia, facial emotion recognition, disease duration, negative-positive symptoms, visual processing beginning stage, facial structure coding stage, expression connotation decoding stage

## Abstract

Deficits in facial emotion recognition are one of the most common cognitive impairments, and they have been extensively studied in various psychiatric disorders, especially in schizophrenia. However, there is still a lack of conclusive evidence about the factors associated with schizophrenia and impairment at each stage of the disease, which poses a challenge to the clinical management of patients. Based on this, we summarize facial emotion cognition among patients with schizophrenia, introduce the internationally recognized Bruce–Young face recognition model, and review the behavioral and event-related potential studies on the recognition of emotions at each stage of the face recognition process, including suggestions for the future direction of clinical research to explore the underlying mechanisms of schizophrenia.

## Introduction

Many studies (1) demonstrate that there is an inextricable relationship between social cognition and psychotic symptoms. Therefore, improving social cognition research plays an important guiding and empirical role in the work of clinical treatment. Social cognition is defined as the ability to perceive other people's intentions and temperament, and it subsequently guides social activities (2), which is mainly reflected in the cognition of other people's expression, character, relationship, and behavior.

Humans are vision-driven animals, and they often infer the psychological state of others as a function of their facial expressions. This is because facial expressions can convey the emotional states of the self and others and also because they can affect the generation and regulation of emotional states and behaviors in response to these signals. Even though there is some debate about how many major or discrete facial expressions there are, Ekman (3) primarily describes six, often referred to as the six “basic” emotions, namely sadness, happiness, surprise, anger, disgust, and fear. Ekman's research (4) indicates that many people can recognize the facial expressions corresponding to these emotions. Deficits and biases in facial emotion recognition are demonstrated to be linked to impairments social and emotional function (5), which aggravate the development of mental disorders (6, 7) and have a negative impact on treatment (8). A variety of mental disorders are characterized by deficits in facial emotional recognition, including schizophrenia (9), autism (10), and depression (11).

Schizophrenic patients often experience multiple dysfunctions in thinking and behavior as well as discord in mental activities (12). Patients often show various clinical symptoms, including difficulties in judging other people's emotions and intentions during the onset of the disease. Such difficulties promote the onset of psychotic symptoms and, ultimately, lead to significant social dysfunction and reduced capacity to function. Bulgari et al. (13) discovered that patients' lack of ability to accurately recognize facial expression is one reason for aggressive behavior often seen in the disease, and as a result, communication between patients with schizophrenia and society is inseparable from the process of recognizing facial emotions. The ability to deal with facial emotions is the central component and deciding factor affecting patients' social cognition, and it is also an important factor affecting social function.

In this paper, we systematically review the behavioral and neuroimaging mechanisms underlying facial emotion recognition in patients with schizophrenia, which has important implications for the development of existing and novel cognitive-behavioral interventions.

## Methods

### Search Strategy and Selection of Papers

A literature search was conducted through the PRISMA guidelines. We searched PubMed and GeenMedical publications from 1976 to 2020 and included the following key phrases: “schizophrenia,” “negative symptoms,” “positive symptoms,” “course of disease,” “face recognition,” and “emotion recognition.”

Patients were included if they were diagnosed with schizophrenia based on diagnostic criteria. Included studies were consistent with (i) publishing raw data in English in peer-reviewed journals and reporting data results using statistical analysis, (ii) including patients with early-onset or first-episode schizophrenia, (iii) including normal controls, (iv) using a face-to-facial emotion recognition task, and (v) using the PANSS scale for symptom assessment. Research on organic mental disorders, substance abuse, and experimental studies; non-English papers; conference abstracts; and book chapters were all excluded. The main contents of the study were title, abstract, and text. Only papers in English were included. The authors screened the available references to determine which would be included in this review for discussion.

**Figure 1:**
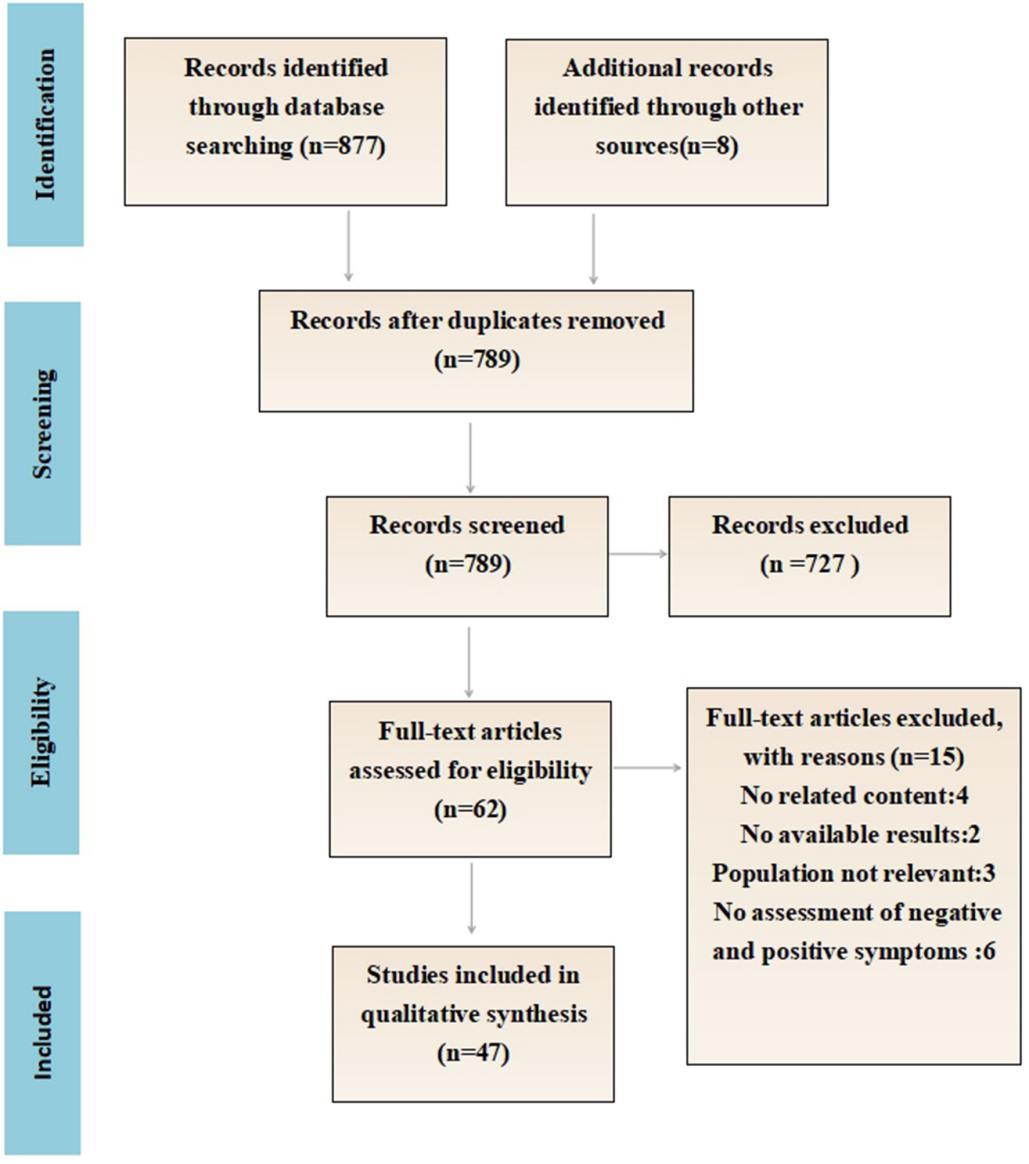
Search strategy and result.

Figure 1 details the flow chart of the eligibility process for this review. A total of 877 articles were identified, including eight additional papers identified through a manual reference search. Following the removal of all of the duplicates, 798 papers were screened manually (title and abstract). Of those, 62 papers were read in full and assessed for eligibility. Finally, 47 papers fulfilled the inclusion criteria. In total, 16 papers explained the difference between the positive and negative emotion recognition ability of schizophrenia, and 11 papers proposed the relationship between clinical symptoms and the facial emotion recognition ability of schizophrenia. Twenty-four papers compared the behavioral differences of facial emotion recognition between schizophrenics and normal people, and nine papers further compared the differences of EEG components.

#### Diagnosis

There were five studies conducted only on schizophrenia samples (14–18). One study was conducted with only normal subjects (19). Nineteen studies compared schizophrenia with normal controls (20–38). One study compared chronic schizophrenia to a health sample (39). Five articles compared schizophrenia, schizoaffective disorder, and healthy controls (40–44). Two studies compared schizophrenia, borderline personality disorder, and healthy controls (45, 46). Two papers compared first-episode psychosis, multiple episodes of schizophrenia, and healthy controls (9, 47). One paper investigated individuals “at risk” for psychosis who met criteria for a programmable state, individuals early in the course of a schizophrenia spectrum illness (SSI), individuals with a chronic SSI, and healthy controls (48). Furthermore, two studies compared schizophrenia, bipolar disorder, and healthy controls (49, 50).

#### Sampling

Eleven studies investigated hospitalized patients (18, 21, 25, 26, 30, 31, 38, 39, 45, 47, 49). Eleven studies surveyed outpatients (14–16, 20, 27, 32–35, 37, 40). Seven papers examined both inpatients and outpatients (9, 17, 22, 24, 44, 48, 50). Nine studies did not provide the source of samples (19, 23, 28, 29, 36, 41–43, 46).

#### Study Design

A cross-sectional design was used in all of the papers. In addition, with the exception of one paper that used the visual search paradigm (23), the others used similar facial emotion recognition tasks.

### Outcome Measures

The main outcome measures were focused on the ability to identify the three stages of the facial emotion recognition process and the different stimuli of facial emotion applied in schizophrenia with no additional methods of analysis. The findings are summarized in the following narrative synthesis.

## Results

### Facial Emotion Cognition Among Patients With Schizophrenia

#### Different Stimuli Patterns of Facial Emotion Applied in Schizophrenia

Turetsky et al. (20) found differences between the cognitive impairment of positive and negative emotions in schizophrenia such that cognitive impairment in facial emotion was much higher in schizophrenia compared with healthy controls and patients with other mental disorders. A large number of studies in recent years (19, 51, 52) have similarly found shorter response times and higher accuracy in recognizing happy faces, a phenomenon that has been referred to as the “advantage effect” in the recognition of happy faces. Some studies (53) show that schizophrenia patients have some obstacles in recognizing negative emotions but that the cognitive processing of positive emotions is relatively similar to levels observed in healthy controls. Emotional deficits in schizophrenia manifest themselves primarily through the recognition of negative emotions, such as sadness, fear, and anger (44). After studying South African Xhosa people, Lepp et al. (37) found that the accuracy of schizophrenics in identifying negative emotional faces, especially high-intensity, negative emotional faces, was significantly lower than that of healthy controls and that they also made many errors in identifying neutral emotional faces. Kohler et al. (54) conducted a meta-analysis and found that facial emotion recognition and discrimination abilities in schizophrenic patients were worse than that of non-psychotic controls and were specific to negative emotions, such as fear and anger. This suggests that patients with schizophrenia have more difficulty identifying negative emotional facial expressions.

However, the literature is inconsistent in identifying defects in neutral and angry faces. Romero-Ferreiro et al. (15) found that schizophrenia impairs the ability to recognize fear. Allott et al. (46) suggests deficits in fear recognition may be a genetic characteristic of schizophrenia and is more representative in negative expressions. Catalan et al. (45) reports a significant difference between patients with first-episode psychosis and healthy controls in recognizing neutral and angry faces, and Barkl's meta-analysis (55) shows no between-group mean differences in anger recognition in a first-episode psychosis sample compared with healthy controls. A further study by Leitman et al. (40) examines processing abnormalities related to fear in schizophrenia, and suggests that schizophrenia impacts some specific social outcomes that are especially linked to danger and risk. Moreover, Schneider et al. (21) found that patients could not distinguish between happy and sad facial expressions and they improperly identified the target emotion as sad. Patients in this study also showed a lack of recognition of happy facial expressions compared with healthy people; Hargreaves et al. (22) and Yildirim et al. (16) arrive at a similar conclusion.

Thus, most studies have observed that schizophrenia patients are specifically impaired in negative emotion recognition, but some studies have yielded inconsistent results. The reason for this controversy may be related to the facial emotion recognition task due to cross-study variations in ease, stimulus type, and presentation. However, different interpretations of the six basic expressions may lead to more inconclusive results, which may also be a key factor affecting patient recognition; further exploration is needed to resolve this issue.

#### Relationship to Clinical Characteristics

Some behavioral studies (9, 14, 48) found that facial processing deficits are similar in both recent-onset and chronic schizophrenia. Specifically, there are deficits in negative emotion recognition in both first-episode and multiepisode patients, suggesting that effect sizes for neural correlates of face processing show comparable stability across different phases of the disease. Moreover, the deficit remains stable over the course of illness, fitting the pattern of a vulnerability indicator as opposed to an indicator of chronicity or severity. Facial emotion recognition is also found to be associated with negative symptoms of schizophrenia (41, 56). Some researchers (23) have also used a visual search paradigm and found that first-episode schizophrenia (FSZ) patients' ability to recognize happy facial expressions correlates with negative symptoms, and patients with severe negative symptoms have poorer facial emotion perception. Indeed, Kitoko et al. (17) also used facial emotion recognition to establish an association between patient deficits and severity of negative symptoms. In addition, Romero-Ferreiro et al. (15) conclude that a deficiency in fear recognition among patients in the first episode was positively associated with negative symptoms. However, some studies (24) find that facial emotion recognition is associated with positive symptoms such as delirium. This suggests that positive symptoms early in the illness may partly explain the deficiency in facial emotional recognition. A more recent study (47) explores differences in facial emotion recognition between patients with first-episode psychosis, multiple-episode schizophrenia, and healthy controls and concludes that accurate recognition of negative emotions is negatively correlated with positive symptoms and further exacerbated with the onset of multiple-episode schizophrenia. In addition, Weiss et al. (18) found that the accuracy of facial emotion recognition in schizophrenia, particularly angry facial emotions, is negatively correlated with the duration of the disease, suggesting that the impairment of facial emotion recognition tends to worsen as the disease progresses.

Facial emotion recognition deficits are stable at each stage of the schizophrenia course and increase with the number of episodes. The reason for the inconsistent results may be that the patients included in the study are not homogeneous, and interpatient differences are greatly affected by the course of the disease, antipsychotic drug use, and other factors.

### Cognitive Stages of Facial Emotion Recognition Processing

**Figure 2:**
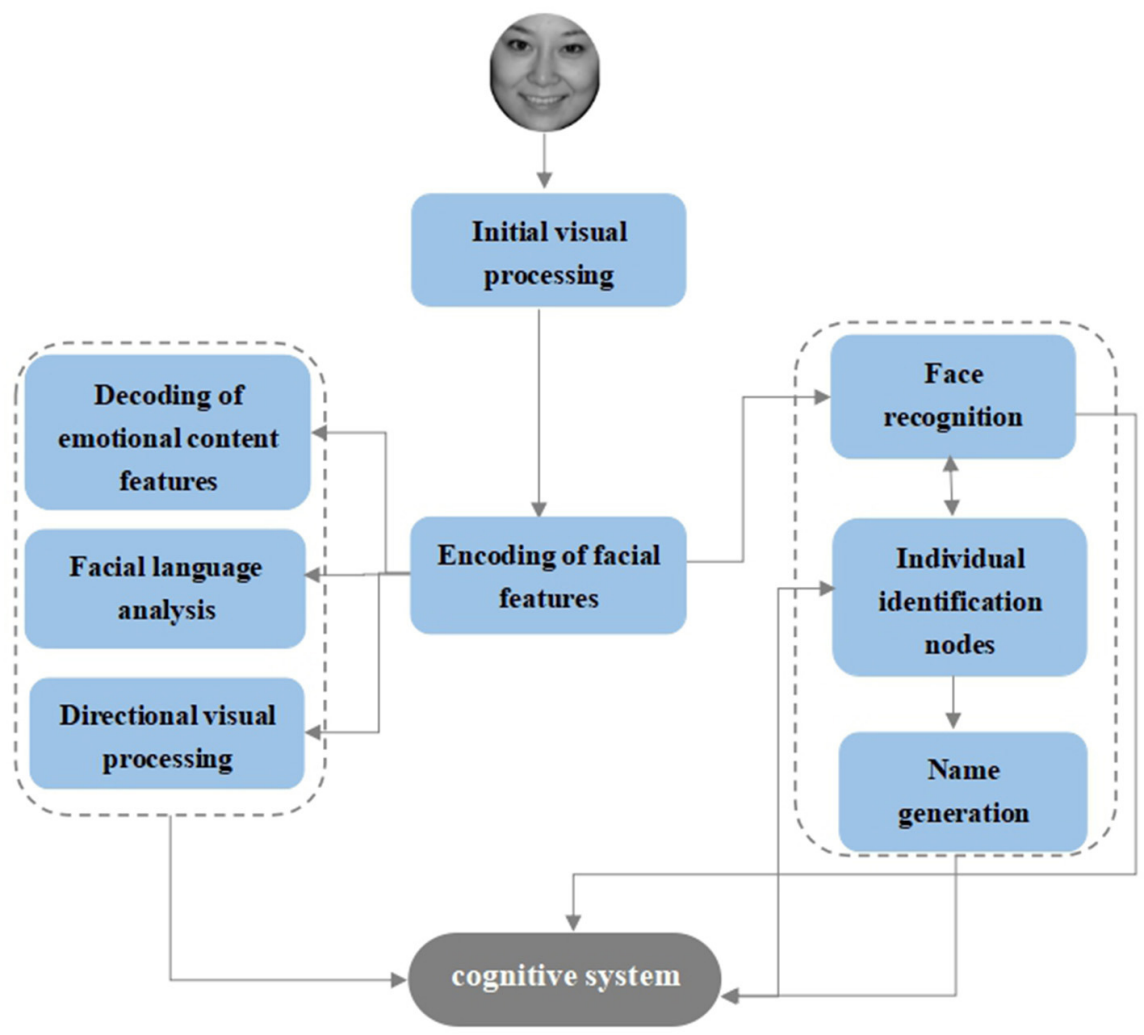
Bruce and Young's Facial Recognition Model.

Bruce and Young's (57) “multistage” cognitive model, also known as the “box and arrow” model, is a theoretical model of face recognition that still has a broad and profound impact today. After the facial stimuli enter the initial phase of visual processing, the next step is to enter the encoding phase of the facial structure, which is followed by two separate channels. The first channel enters the feature processing of facial information and contains four parallel processing units: decoding, analysis of expressive features, face language analysis, and direct visual processing, and the second channel is related to face recognition and contains three serial processing units: the face recognition unit, individual feature unit, and name generation unit (Figure 2). According to this model, the process of facial emotion recognition can be divided into three stages: the initial stage of visual processing, the encoding stage of facial features, and the decoding stage of facial expression connotation.

Schizophrenia shows widespread impairments in emotional facial expression recognition and processing (25), but the question remains as to whether the patient's clinically manifested deficits in facial emotion recognition reflect a single impairment of the ability to decode stages of mood or whether it is a secondary manifestation of impairments in facial or general visual perception. The answer depends on the use of techniques that allow us to observe the underlying cognitive mechanisms with precision in the temporal dimension. Due to the high temporal resolution of the event-related potential (ERP) technique, a growing number of studies have used it to assess facial expression recognition in schizophrenic patients. Three have been primarily identified: P100 for detecting basic visual processes (triggered by non-facial physical clues), N170 for detecting facial feature encoding (triggered by the information-containing faces), and N250 for detecting expression decoding (triggered by information-containing emotions) were used to explore the various stages of facial emotion processes in schizophrenia (58).

#### Visual Processing Beginning Stage

It has been shown that people with schizophrenia have difficulty integrating and organizing visual information through a top-down process, i.e., the inability to combine discrete elements to form a holistic representation. One review (59) mentions that, at the behavioral level, visual perceptual deficits in schizophrenia are not specific to faces but also to objects, are most often present when the cognitive (and perceptual) demands of the task are important, and get worse in the course of the illness. Thomas et al. (26) explore whether patients with schizophrenia exhibit generalized perceptual deficits or specific face-processing deficits and find that patients have poor accuracy and long reaction times to both human and non-human faces. This implies that patients have a general visual processing deficit, a general perceptual disability, but no face-specific treatment deficits. However, Silverstein et al. (27) use pictorial materials in which patients were asked to make a choice response and conclude that early impaired processing of global information diminishes the information reaching the fusiform face area, resulting in insufficient signal strength to process facial stimuli. They further conclude that, in addition to a decrease in attention, more general perceptual difficulties can highlight deficits in the treatment of the face. Indeed, Dark et al. (60) pose the question of whether schizophrenics are impaired in processing non-emotional aspects of the face, concluding that, in addition to a decrease in attention, more general perceptual difficulties can highlight deficits in facial perception.

Neurophysiological evidence suggests there are altered levels of early visual processing, which may be linked to defects in the interaction of large cell and parallel pathways affecting subsequent information processing. Doniger et al. (39) assess schizophrenic patients' ability to recognize intact objects from fragmentary information, a process known as perceptual closure. The perceptual closing process is indexed by the closing negativity (Ncl), a recently defined component of the ERP that appears in the visual cortex. This study evaluates the neural integrity of the perceptual closing process in schizophrenia by observing the generation and performance of Ncl. Patients with schizophrenia were found to be profoundly impaired in perceptual closure as indicated by both impaired performance and impaired Ncl generation. Kim's team (28) further found that deficits in the visual pathway dependent on spatial frequency can lead to anomalies in facial emotion recognition in schizophrenia, showing that, compared with the healthy control group, the P100 amplitude of fear to facial images decreased in the low spatial frequency (LSF) but increased in the high spatial frequency (HSF) for neutral face images. Activation of N170 in the right hemisphere was improved with a longer latency in the LSF condition but not in the normal group. This leads to the conclusion that defects of LSF-dependent visual pathways (including large cell neurons) in schizophrenia alter early visual processing, resulting in dysfunctional facial emotional recognition. Thus, schizophrenic patients are functionally impaired before they even begin the coding phase of facial structures.

#### Facial Structure Encoding Stage

In addition, the question of whether patients' emotional recognition deficits are a state or whether they are qualitatively modified has been of great interest in the field. Kerr and Neale (29) matched an emotion perception task with a control task of non-emotional face perception through a specific impairment experimental design, and they found that schizophrenic patients performed worse than healthy controls on both tasks although there was no statistically significant difference between performance on the emotion perception and control task. In the study by Martin et al. (30) that examines the ability of stable schizophrenia patients to match emotions with identity information during changes in emotion and identity dimensions, they conclude that emotional cognitive deficits were only general perceptual impairments in processing face information rather than an emotion-specific impairment. Bauser et al. (42) arrive at a similar conclusion and suggest that this could be due to a dysfunction in the treatment of information about facial structures in schizophrenia. Similar studies (31, 50) suggest that the impairment in schizophrenic facial emotion perception is general rather than specific as researchers find that the impairment is likely to be influenced by the integrative effects of emotion and cognition associated with impairments in goal setting, motivation, memory, and attention. Behavioral studies (59) of non-emotional perception also show that schizophrenic patients and healthy controls have difficulty matching and identifying individuals on age and gender recognition tasks in the former such that patients also have impairments on non-emotional face tasks. Thus, facial recognition deficits and emotions present in schizophrenia patients may be general facial perception deficits rather than specific ones. In addition, several studies (32, 61) found deficits in both face recognition and facial emotion recognition in schizophrenic patients and concluded that there may be an interaction between the two.

Previous studies (62) also report deficiencies on the N170 in facial expression recognition tasks in schizophrenia. In one study investigating schizophrenia, bipolar disorder, and healthy controls, researchers found that schizophrenia patients' ability to recognize emotions, such as happiness, anger, fear, shame, sadness, and surprise, were significantly lower compared with healthy subjects, resulting in abnormalities their ability to encode facial structures and to recognize a variety of basic expressions (49). Brenner et al. (43) used a delayed-match sample task to explore the three EEG components of P100, N170, and N250 in schizophrenia and schizoaffective disorder and normal individuals; N170 was found to be significantly longer in the patient group compared with the normal group. Maher et al. (33) equalized the visual contrast signals between the schizophrenia and face pictures and trees according to the individual perceptual abilities of the patient and normal groups, and in the normal group, the N170 amplitude induced by face images was significantly higher in all three tests compared with the normal group. Trees, however, did not differ significantly in N170 amplitude between faces in patients, suggesting that the mechanisms responsible for facial processing in the schizophrenic brain lack selectivity in the temporal response. This also suggests that the ability to encode facial structures is impaired in schizophrenia. Lahera et al. (32) further found that schizophrenia patients are also less precise on the familiarity task—results that the authors linked to a general deficit in facial processing present in both visual perception (P100) and face perception (N170), i.e., the interaction of the face and vision. This suggests that the onset of visual processing and the encoding phase of face structure interact with each other and that facial recognition deficits prevalent in schizophrenia may be the results of perceptual deficits in basic visual information processing. These results support the idea that facial perception is caused by broader deficits in visual perception and other common cognitive deficits in schizophrenia.

#### Expression Connotation Decoding Stage

There are also some researchers who hold the view that patients are impaired specific to emotions. Behere et al. (38) obtained results to suggest that schizophrenic patients performed worse in recognizing facial expressions compared with health controls but not in classifying ambiguous faces, suggesting that patients are deficient in facial emotion recognition but otherwise have intact facial perceptual processing. Similarly, Kosmidis et al. (34) show that patients in recovery have deficits in an emotional matching task and that the cause of this deficit is a perceptual impairment of emotional meaning rather than an impairment secondary to visual perception.

A classic experiment by Jonathan et al. (35) evaluated the three ERP components to explore various stages of facial affective processes in schizophrenia. Subjects were asked to identify the gender of a face, a facial expression, and a building with one or two stories. The intact P100 and N170 patterns in the results indicate that patients have intact basic visual processing and facial feature encoding abilities although the abnormal N250 indicates that schizophrenic patients are less efficient at decoding facial affective features. However, because patients are on medication, results may be influenced by the medication, and tasks may be confounded accordingly. Similar results were obtained in a recent ERP experiment by Sandhya et al. (36), which found that N250 latencies were significantly longer in the patient group compared with those in the normal group for all four emotions. However, they found no significant differences in the mean amplitude and peak latency of the N170 waveform between the two groups, suggesting that the ability to decode facial expressions is significantly impaired in schizophrenia. However, this study had a small sample size and low power, making the need for future research into this hypothesis necessary.

**Figure 3:**
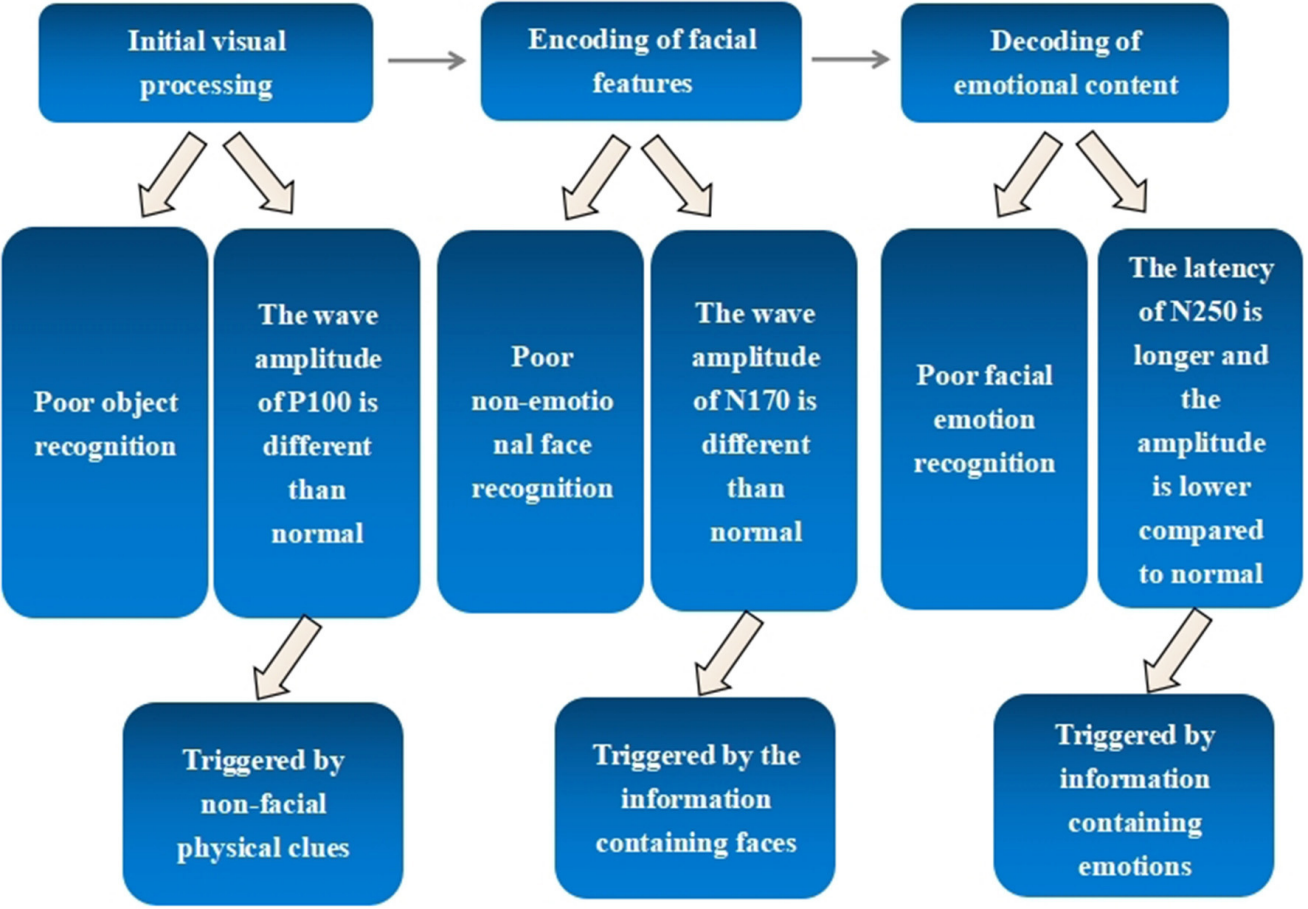
Behavioral abilities and corresponding ERP components in three stages of facial emotion recognition processing in schizophrenia.

The behaviors and corresponding ERP components in three stages of facial emotion recognition processing in schizophrenia are shown in Figure 3.

## Discussion

In summary, studies on schizophrenia in facial emotion recognition have focused on differences in positive and negative emotion recognition and their relationship to psychotic symptoms. Most current studies use a facial emotion recognition task, but the specific stimuli and the form of the task differ across studies. Some use picture stimuli that are both static and dynamic, and others ask participants to directly identify the emotions expressed by the stimuli. Some studies ask participants to classify the emotions expressed by the stimuli as positive or negative, and others ask participants to rate the intensity of the emotions expressed by the stimuli. Such variability in tasks may account for the differential outcomes in patients' recognition of positive and negative emotions. However, most studies find that patients are more impaired in the recognition of negative emotions, leading to the wider use of only negative emotions as stimulus material, making the results unbalanced. Therefore, future experimental studies should include all six basic emotions.

A considerable number of articles find correlations between face recognition and negative symptoms, which is in accordance with a recent meta-analysis reporting a moderate effect size relationship [(*r* = −0.22; (61)]. A few studies also conclude that the two are positively correlated. Some researchers (63) find that anhedonia and emotional blunting may be more associated with impaired emotional recognition than other negative symptoms; it is, therefore, not difficult to conclude that more severe negative symptoms result in greater patient deficits. Such findings also reflect the profound impact of negative symptoms on face-emotion recognition and even social functioning.

Studies confirm that impairment of facial emotional recognition is present from the onset of schizophrenia, in both recent episodes and chronic schizophrenia, and is further exacerbated with multiple episodes of psychosis. However, it may depend on or contribute to the development of clinical symptoms. Hence, the evaluation of first-episode schizophrenia should be included in the clinical evaluation and the treatment plan from the very beginning of the disease to achieve early detection, early intervention, and early treatment for patients. Schizophrenia is closely related to clinical symptoms, and this is a topic that cannot be neglected in future research in this field.

Most studies show that patients are impaired in all three stages of facial emotion recognition processing and that there is an interaction between the visual onset processing and face encoding stages and between the face encoding and emotion decoding stages. Moreover, the evidence for emotional specificity of schizophrenic facial emotion recognition is insufficient and requires further research. Emotion recognition may be general perceptual rather than emotion-specific, and clinically corrective treatment should be considered starting with learning reinforcement for object and face recognition, followed by training in emotion recognition, which may be more effective. Finally, several studies confirm that the P100, N170, and N250 responses in facial emotional tasks of patients are different from those of healthy controls. Among them, N170 and N250 show longer or no differences in latency compared with controls, which may be caused by inconsistencies in the selection of patient groups and small sample sizes. In general, however, the current studies are similar in that they all use a picture recognition task with static stimuli and are mostly limited to three independent stages of damage. As such, there is little research on the connection between stages and lack of a complete, comprehensive, and coherent account of the mechanisms underlying face information transmission.

Current studies on the three stages of facial emotion recognition in schizophrenia are mostly single-center studies. A multicenter clinical trial could be considered to expand the existing sample size, further explore the patient's processing mechanism, and enhance the persuasive power. Moreover, most of the previous studies have used photographs of real people as experimental materials. Future studies should consider designing the materials to be more ecological, such as dynamic pictures of real people and small videos with contextual backgrounds to create stimuli as close to life as possible for patients so that they can be more realistic and accurate for basic research or clinically targeted treatments. In addition, can impairments in the connection process between each stage also lead to recognition barriers? This requires experimental materials that can realistically isolate each of the information transfer processes to be studied, and it seems that animated materials can more accurately represent the transfer process from stage to stage. However, no such study has been done so far, and this can be further explored in future studies.

The heterogeneous findings regarding the recognition of facial emotions in schizophrenia reflect the complexity of this disorder. Although impairments in facial expression recognition have been reported in the research, there is still a need to determine the underlying neural mechanisms. Furthermore, it is necessary to conduct ecological studies to determine the impact of these deficits on social interactions, and in this regard, virtual reality technology can be used to design specific social scenarios that can be explored in a simulated environment to develop effective rehabilitation measures for the possible impact of these deficits on daily life.

## Data Availability Statement

The original contributions presented in the study are included in the article/supplementary material, further inquiries can be directed to the corresponding authors.

## Author Contributions

YX designed and supervised the study. ZG drafted the manuscript. WZ, SL, ZL, and CY collected some literature. All authors contributed to the article and approved the submitted version.

## Conflict of Interest

The authors declare that the research was conducted in the absence of any commercial or financial relationships that could be construed as a potential conflict of interest.
